# Positive Psychology Broadens Readers’ Attentional Scope During L2 Reading: Evidence From Eye Movements

**DOI:** 10.3389/fpsyg.2019.02245

**Published:** 2019-10-04

**Authors:** Chi Yui Leung, Hitoshi Mikami, Lisa Yoshikawa

**Affiliations:** ^1^Department of Economics, Nagoya Gakuin University, Nagoya, Japan; ^2^Department of English Language and Culture, Chubu University, Kasugai, Japan; ^3^Institute of Liberal Arts and Sciences, Toyohashi University of Technology, Toyohashi, Japan

**Keywords:** L2 reading, self-efficacy, positive psychology, reading strategy, first-fixation location, eye movement

## Abstract

While positive psychology has drawn increasing interests among researchers in the second language (L2) acquisition literature recently, little is known with respect to the relationship between positive psychology and mental processes during L2 reading. To bridge the gap, the present study investigated whether and how positive psychology (self-efficacy) influences word reading strategies during L2 sentence reading. Based on previous studies, eye-movement patterns with first-fixation locations closer to the beginning of a word can be characterized as an attempt to process the word with a *local strategy*, whereas first-fixation locations farther away from the beginning and closer to the center of a word can be considered as an attempt to use a *global strategy*. Eye movements of a group of Japanese learners of English (*N* = 59) were monitored, and L2 reading self-efficacy was used to assess the participants’ positive belief toward their L2 reading skills. Based on Fredrickson’s (1998) broaden-and-build theory, we predicted an effect of L2 reading self-efficacy on participants’ first-fixation locations. Results from mixed-effects regression showed that while reading strategies depended in part on other factors such as L2 reading proficiency and word properties, L2 self-efficacy influenced reading strategy. The present data suggest that while more self-efficacious L2 readers prefer a more efficient global strategy, attempting to read the word as a whole word, less self-efficacious L2 readers tend to employ a local strategy, focusing more on sublexical information. These findings lend support to the broaden-and-build theory in the context of L2 processing. The present study has implications for how positive psychology works along with L2 proficiency in the development of strategic selection during reading.

## Introduction

While negative emotions, predominantly anxiety, have been the center of research interest in the second language (L2) acquisition literature until recently, researchers have also shed light on the role of positive psychology, arguing for a more comprehensive perspective on the dimensions of emotions that encompasses the effects of both negative and positive emotions on L2 learning (e.g., MacIntyre and Gregersen, 2012a, b; Dewaele and MacIntyre, 2014; MacIntyre and Mercer, 2014). One early study, from Lake (2013), reported that measures for L2-related positive psychology and L2 English proficiency were positively correlated among Japanese college and university students, a finding supported by subsequent studies focusing on foreign language enjoyment in the classroom (Dewaele and Alfawzan, 2018; Saito et al., 2018), suggesting a beneficial effect of positive psychology on the development of L2.

Apart from the link between positive psychology and L2 proficiency/L2 test performance, some studies have also focused on the relation between positive psychology and L2 behavior, reporting that foreign language enjoyment enhanced L2 learners’ willingness to communicate in the classroom (Dewaele and Dewaele, 2018; Khajavy et al., 2018). These findings are in line with Fredrickson’s (2001) notions on the function of positive emotions to broaden people’s thought-action repertoires (see Khajavy et al., 2018). The present study aimed to build on the notions of Fredrickson (1998, 2001, 2003, 2004) with respect to the effects of positive psychology on L2 behavior, focusing on the mental processes underlying real-time L2 reading behavior, which, to date, has drawn comparatively little attention in the literature of L2 acquisition.

### Positive Psychology and Scope of Attention

Fredrickson’s (1998, 2001, 2003, 2004) work has inspired further research on positive psychology among researchers in the field of L2 acquisition (e.g., MacIntyre and Gregersen, 2012a, b; Dewaele and MacIntyre, 2014). Building on the earlier studies of Isen and colleagues on how emotions are associated with cognition (e.g., Isen and Daubman, 1984; Isen et al., 1985; Isen, 1990; see Fredrickson, 2004), Fredrickson (2001) proposes a “broaden-and-build” theory, suggesting that while negative emotions tend to “narrow people’s attention, making them miss the forest for the trees” (p. 221), positive emotions such as joy, interest, contentment, and love, can “broaden people’s momentary thought-action repertoires and build their enduring personal resources, ranging from physical and intellectual resources to social and psychological resources” (p. 219).

In two experiments, Fredrickson and Branigan (2005) tested the broaden-and-build theory’s *broaden hypothesis*, which “states that positive emotions broaden the scopes of attention, cognition, and action, widening the array of percepts, thoughts, and actions presently in mind” (p. 315). American university students viewed films eliciting positive emotions including amusement and contentment, negative emotions including anger and anxiety, or neutral emotions (in a control condition). The participants then underwent a global-local visual processing task developed by Kimchi and Palmer (1982), in which they were presented with global-local figures (e.g., a triangle made up of square elements and a triangle made up of triangular elements), and were instructed to judge the similarity among figures. Using this method, the extent to which participants were influenced by the global or the local features of the figures was used to assess whether the scope of participants’ attention was broadened, narrowed, or remained the same. The results support Fredrickson and Branigan’s (2005) hypothesis that positive emotions broaden the scope of attention, a finding further backed by later studies (Wadlinger and Isaacowitz, 2006; Rowe et al., 2007), which suggest that positive emotions draw more attention to global than local features during information processing (cf. Sung and Yih, 2016).

With respect to L2 studies, it has been generally acknowledged that psychological variables and emotions are associated with cognition; however, related studies have focused on oral communication (e.g., Jeong et al., 2016). While there has been a line of research on L2 and bilingual processing of emotional words and text (e.g., Eilola et al., 2007; Hsu et al., 2015; Iacozza et al., 2017), research on how emotional or psychological states influence the scope of attention during L2 processing for reading has been limited. An early eye-tracking study from Dizney et al. (1969) reported that more anxious native English readers tended to make more fixations during reading. A more recent eye-tracking study on processing of emotional words conducted by Knickerbocker et al. (2015) showed that native English readers’ levels of depression and anxiety, as assessed by questionnaire items, influenced where readers’ eyes moved during sentence reading (in experiment 2). When moving their eyes from a word to the next, upcoming word, readers showing higher levels of depression and anxiety tended to initially (as a *first-fixation location*) move their eyes to the upcoming word at a position closer to the beginning of the word, compared to those showing lower levels of negative emotions. While the underlying mechanism of such effects of negative emotions on first-fixation locations^1^ was not explicitly discussed by Knickerbocker et al. (2015), other recent studies (e.g., de León Rodríguez et al., 2015, 2016) have associated eye-movement measures including first-fixation locations with the *reading strategy* applied to words. This line of study may provide us a potential basis for how to investigate the influence of positive psychology our mental processes during L2 reading, and will be further discussed in the following section.

### Reading Strategies, Eye Movements and First-Fixation Locations

According to the dual-route word recognition model (Coltheart et al., 2001), written words are processed via two routes: a *sublexical route* and a *lexical route*. Through the sublexical route, the letters of a word are translated from graphemes, whether single letters or sequences of letters, into phonemes, in a local and serial manner (i.e., following grapheme–phoneme conversion rules), before the word meaning is accessed. For instance, when THIGH is processed via the sublexical route^2^, two graphemes, TH and IGH, are converted into /θ/ and /ai/, to assemble the phonological form of the word. In contrast, through the lexical route, the letter-string of a word is processed in a holistic (whole-word), parallel manner based on lexical knowledge. Readers directly access the word’s orthography, as well as other lexical semantic information, in the mental lexicon via this route from the word’s written form. Whether a word is processed via the sublexical or the lexical route depends on word properties such as length and frequency (Tiffin-Richards and Schroeder, 2015).

Recent studies have analyzed the dual-route perspective on word processing based on eye movements during reading (Hawelka et al., 2010; Kuperman and Van Dyke, 2011; Rau et al., 2014, 2015; de León Rodríguez et al., 2015, 2016; Gagl et al., 2015; Tiffin-Richards and Schroeder, 2015), and some of these eye-tracking studies have reported that readers’ first-fixation location on a word may reflect their preference for the lexical or sublexical route during word reading (Hawelka et al., 2010; Kuperman and Van Dyke, 2011; de León Rodríguez et al., 2015, 2016). That is, readers will attempt to adopt a *local strategy* when processing a word via the sublexical route, with first-fixation locations closer to the beginning of the word, while a *global strategy* will be employed to process a word through the lexical route, with first-fixation locations farther away from the beginning and closer to the center of a word (de León Rodríguez et al., 2015, 2016).

First-fixation locations on words have drawn interests in research on eye movements in reading (see Rayner, 1998, 2009). During reading, the *optimal viewing position* for a word is around the center of the word (Vitu et al., 1990; O’Regan and Jacobs, 1992), as in this position most letters fall into the range of highest visual acuity. First-fixation locations farther away from the center of a word tend to lead to refixations on the word (O’Regan, 1990; Rayner et al., 1996).

Word properties, predominantly word length, have also been major factors influencing first-fixation location (Rayner, 1979; Joseph et al., 2009), and hence, reading strategy (Hawelka et al., 2010; Kuperman and Van Dyke, 2011). In addition, individual differences resulting from different levels of reading skills have also been reported to have an effect (Kuperman and Van Dyke, 2011). Based on eye movements during sentence reading among native English readers, Kuperman and Van Dyke (2011) reported that first-fixation location is a function not only of word length and frequency but are also of individual differences in reading skills, such as word decoding skills. Their findings suggest that a global strategy represented by first-fixation locations closer to the center of a word is more preferred for shorter and higher frequency words and among readers of higher reading skills. For L2 readers, a recent bilingual study by de León Rodríguez et al. (2016) has reported that bilingual readers tend to favor a local strategy when reading in their (non-dominant) L2, indicating a proficiency effect on first-fixation location and word reading strategy.

To sum up, the current state of the literature with respect to eye movements during reading has shown that first-fixation locations, which can be interpreted as indicating preferences for reading strategies, are a function of word properties and reading abilities. While aforementioned studies such as Knickerbocker et al. (2015) have reported data suggesting that affective or emotional factors may also influence first-fixation locations, their data are limited to negative emotions such as anxiety. More importantly, the theoretical account of how the emotions of a reader influence first-fixation location is yet to be explored.

### The Present Study

In the literature on L2 acquisition, as noted earlier, while positive psychology has drawn increasing interests among researchers, no studies have investigated whether and how positive psychology influences mental processes during L2 reading. The present study aimed to bridge the gap by employing (1) Fredrickson’s (1998, 2001, 2003, 2004) broaden-and-build theory, and (2) the aforementioned dichotomous conceptualization of eye-movement patterns based on the dual-route word recognition model (Coltheart et al., 2001) as the theoretical basis to examine whether and how L2 readers’ positive emotions toward L2 reading influence their L2 reading strategies. According to the broaden-and-build theory’s broaden hypothesis (Fredrickson and Branigan, 2005), which predicts an effect of positive emotions on attentional scope, we expect that readers who are emotionally more positive toward their L2 abilities should attend more to the global features of words (i.e., word orthography and lexical information) instead of local features (i.e., graphemes and sublexical information), and hence should prefer a global to a local strategy during L2 reading. In terms of eye movements, based on previous findings (e.g., Hawelka et al., 2010; de León Rodríguez et al., 2015, 2016), we anticipate that readers who favor a global strategy will attempt to fixate closer to the center of a word, while readers preferring a local strategy should have more first fixations closer to the beginning of a word.

Methodologically, in the present study, we used L2 reading self-efficacy, defined as a positive belief in one’s own problem solving and L2 reading skills, to index positive psychology. Self-efficacy comes from one’s experiences and emotional states (Bandura, 1997; Maddux, 2009). Thus, successful and positive experiences and feelings enhance self-efficacy. In the context of SLA, self-efficacy has been reported to have positive relationships with L2 reading and listening proficiency (Mills et al., 2006; Lake, 2013; Yabukoshi, 2018), learning strategies for L2 oral and pronunciation learning (Yang, 1999; Sardegna et al., 2018), as well as L2 vocabulary learning (Mizumoto, 2012, 2013), but little is solidly known with respect to how self-efficacy is associated with language processing during real-time L2 behavior. Based on the broaden-and-build theory, we predict that more self-efficacious L2 readers will prefer a global strategy during L2 reading.

We monitored L2 reading processes based on eye movements in a group of Japanese learners of English undergoing a sentence reading task to validate our prediction. As pointed out earlier, global vs. local reading strategy use is influenced by word properties such as word length and word frequency, as well as language proficiency (Hawelka et al., 2010; Kuperman and Van Dyke, 2011; de León Rodríguez et al., 2016). We anticipate interactions among all these factors and self-efficacy on reading strategies, indexed by first-fixation locations. For instance, self-efficacy and proficiency of L2 readers may matter little for first-fixation location when reading short words, most readers tend to fixate closer to the center of short words (e.g., Hawelka et al., 2010; Kuperman and Van Dyke, 2011). Moreover, self-efficacy may also interact with proficiency, as self-efficacy can only come into effect given “requisite skills and knowledge” (Schunk and Pajares, 2002, p. 16).

The specific research questions of the present study are as follows:

(1) Does L2 reading self-efficacy influence L2 reading strategies?
(2) How is L2 reading self-efficacy associated with L2 proficiency and word properties with respect to effects on reading strategies?

## Materials and Methods

### Participants

Fifty-nine Japanese native speakers (36 females and 23 males), aged 20.46 on average (*SD* = 1.96), received remuneration for participating in the experiment. All participants, with normal or corrected-to-normal vision, were undergraduate or graduate student at a university in Japan. They learned English as a foreign language (EFL), and had undergone at least 6 years of formal English instruction in Japan. Their English proficiency was between intermediate and upper-intermediate, with a mean self-reported TOEFL ITP (Test of English as a Foreign Language – Institutional Testing Program) score of 525 (*SD* = 52). The present study was conducted in accordance with the recommendations of the Grant-in-Aid for Scientific Research of the Japan Society for the Promotion of Science (JSPS). Written consent was collected from each participant.

The reading comprehension (RC) test developed by the Edinburgh Project on Extensive Reading (Hill, 1992) was employed to assess the L2 reading proficiency (L2RC) of the participants. The test consists of a narrative story and 20 questions on the content of the text; during the test, the participants were told to finish answering the questions within 30 min. The mean score on the comprehension test was 16.53 (*SD* = 6.59) and the Cronbach’s alpha of the reading test was 0.86. For the assessment of L2 reading self-efficacy (L2RSE), an L2RSE index was obtained using related question items (e.g., “I am good at reading in English”; *k* = 4), taken from Mori (2002). Items were translated into Japanese and were responded to on a 6-point Likert-type scale (1 = strongly disagree, 6 = strongly agree). The mean rating for the L2RSE index among the participants was 3.59 (*SD* = 0.98), and the Cronbach’s alpha was 0.75.

### Materials

Each participant read 120 English sentences in the eye-tracking experiment. On average, each sentence contained 11.52 words (*SD* = 1.80), with a mean word length of 4.69 letters (*SD* = 2.31) and mean log-transformed HAL word frequency of 12.63 (*SD* = 3.14), as obtained from Balota et al. (2007). Among the 120 sentences, six were directly selected from the stimuli used in Kuperman and Van Dyke (2011). While there are three types of sentences used in Kuperman and Van Dyke (2011) – simple sentences without any embedding, sentences with an embedded relative clause, and sentences with double embeddings – we did not select from sentences with double embedding, as they were deemed too difficult to our L2 participants. Others were either sentences modified from Kuperman and Van Dyke’s (2011) stimuli, or sentences created by a native English speaker. The final stimuli consisted of 45 simple sentences (e.g., “The traffic accident caused some serious injuries”) and 75 sentences with a relative clause (e.g., “The tourist who had a camera took pictures of the mountain”).

### Procedure

All tasks were conducted individually in a sound-proof room. Brief practice sessions until participants got used to the required tasks preceded all of the measures. For the eye-tracking sessions, they sat in front of a 21-inch CRT monitor (EIZO FlexScan T965; 1024 × 768 pixel resolution; refresh rate = 120 Hz) with a chinrest at a viewing distance of approximately 65 cm to record eye-movement data from the participant’s right eye at a sampling rate of 1000 Hz, using an EyeLink 1000 Desktop Mount (SR Research Ltd.) eye-tracker. At that distance, three letters subtended about one degree of the visual angle. Five-point calibration was conducted, and recalibrations were performed before each trial if the calibration became inaccurate.

For each trial, a fixation mark first appeared at the center-left of the computer screen. The participants fixated their eyes on the fixation mark until the mark was replaced by an experimental sentence, with the first letter of the first word at the same position as the fixation mark. Each sentence was displayed in a monospaced font (Courier New), black on a light-gray background, in a single line. The participants were instructed to read each sentence silently and to press a button right after they had finished reading. Presentation of sentences were randomized. One-third of the sentences were followed by a yes/no comprehension question to make sure that the readers had paid attention to the sentences and read them properly; the mean accuracy on these questions was 90.1%.

After the eye-tracking session, the participants took part in the RC test and answered the questionnaire items for L2RC and for L2RSE. The entire experiment was finished within 90 min for each participant.

### Data Treatment and Analysis

Fixations with durations below 80 or over 1000 ms were classified as outliers (5.6% of the data). In addition, the initial and final words of each sentence, words with punctuation, as well as words without word-frequency information based on Balota et al. (2007) were removed from analysis (17.7% of total number of words read by a participant). To assess word reading strategy, first-fixation location (relative fixation position in a word measured as position/word length, with a value of 0.5 representing a first-fixation positioned at the center of the word, and a lower value representing a fixation closer to the beginning of a word), gaze duration (log-transformed during modeling; fixation duration measured as the sum of fixation time on a word before the eyes leave the word)^3^, and first-pass fixation count (number of fixations on a word before the eyes leave the word) were used in the analysis. Instead of refixation probability, which measures the probability a word will be fixated more than once, we used first-pass fixation count since L2 participants tended to fixate more than twice for longer words.

All three of these measures are first-pass reading measures aimed at monitoring early cognitive processes during word processing. The number of observations from the eye-movement data for each measure was 55,467. 33.6% of the words fixated were made with multiple fixations. It is noteworthy that while first-fixation locations closer to the center are regarded as indicating a preference for attempting a global strategy over a local one, this does not necessarily mean that a word is eventually processed via the lexical route. A reader may attempt to adopt a global strategy and target a fixation position closer to the center of a word, in which the likelihood of making refixations is lower (O’Regan, 1990; Rayner et al., 1996), but refixations may still be made when lexical access is not completed with the first fixation (Reichle et al., 2003, 2009). In such a case, multiple fixations are made not only because of the decision taken on reading strategy at the beginning but also because the reader needs more time to process the word. In other words, instead of focusing on how a word is eventually processed, the preference of reading strategy depends on readers’ attempt to decide where to fixate on in an upcoming word – a decision made before the word is fixated on.

Mixed-effects modeling using R (R Core Team, 2016) and the lme4 package (Bates et al., 2015) was employed to analyze the effects of L2RC and L2RSE on these eye- movement measures. Participant and word were treated as random effects. For the predictors, in addition to L2RC and L2RSE, word frequency (WF) and word length (WL) were also entered into the models, as these are factors that influence word reading strategy (e.g., Kuperman and Van Dyke, 2011). Interactions among L2RC, L2RSE, and word properties (WF or WL) were included in the linear mixed-effects models; in addition to these predictors and interaction effects, control predictors including trial order, word position in the sentence, as well as preceding word frequency (PWF) and preceding word length (PWL) were also entered into the modeling as they have been reported to influence eye movements (e.g., Kliegl et al., 2006; Kuperman and Van Dyke, 2011). Nevertheless, the effects of the control predictors will not be discussed as they are not directly related to the interests of the present study. Since L2RSE was correlated with L2RC (*r* = 0.481, *p* < 0.001), a linear model in which L2RSE was predicted by L2RC was fitted, and the residuals of the linear model were used as the predictor of L2RSE in the mixed-effects models, so as to partial out the effects of L2RC. The same procedure was completed for WF and WL, which were strongly correlated (*r* = –0.748, *p* < 0.001), with WF residualized prior modeling. All continuous variables were centered and standardized.

For model selection, we first fitted a maximal model including all the predictors and the aforementioned interactions. Predictors and interactions which did not improve model fit were then removed from modeling in a backward stepwise approach, using the step() function of the ImerTest package (Kuznetsova et al., 2015). This package was also used to calculate the *p*-values of the fixed effects in the linear mixed-effects models (based on Satterthwaite’s approximation). For the visualization of interaction effects among predictors, partial effects were computed with the remef (Hohenstein and Kliegl, 2015) package and were displayed in figures created with the ggplot2 package (Wickham, 2009). Descriptive statistics for the eye-movement measures is presented in Table 1.

**TABLE 1:** Mean (*M*), standard deviation (*SD*), standard error of the mean (*SE*), and 95% confidence interval (*CI*) of the eye-movement measures.

**Eye-movement measures**	***M***	***SD***	***SE***	***CI***
First-fixation location	0.404	0.254	0.001	0.002
Gaze duration (ms)	430	319	1	3
First-pass fixation count	1.522	0.990	0.004	0.008
				

## Results

### First-Fixation Location

The results for the final model fitting first-fixation location are displayed in Table 2. Main effects of WF and WL were significant, indicating that on average, first-fixation locations moved closer to the beginning of a word with a decrease in word frequency or with an increase in word length. Main effects of L2RC and L2RSE were not significant; however, significant interactions involving L2RC, L2RSE, and word properties were observed.

**TABLE 2:** Linear mixed-effects model fitting first-fixation location.

	***b***	***SE***	***t***	***p***
**Fixed effects**				
Intercept	0.4047	0.0064	63.5310	<0.0001
L2RC	0.0122	0.0062	1.9480	0.0565
L2RSE	0.0068	0.0063	1.0770	0.2861
WF	0.0107	0.0017	6.2280	<0.0001
WL	–0.0754	0.0016	–45.7700	<0.0001
L2RSE × WF	0.0023	0.0010	2.3200	0.0204
L2RSE × WL	–0.0021	0.0010	–2.0670	0.0387
L2RC × WF	Removed			
L2RC × WL	0.0014	0.0010	1.4260	0.1538
L2RC × L2RSE	0.0063	0.0067	0.9400	0.3515
L2RC × L2RSE × WF	Removed			
L2RC × L2RSE × WL	0.0023	0.0011	2.1840	0.0290
PWF	–0.0053	0.0012	–4.4160	<0.0001
PWL	0.0297	0.0012	25.0790	<0.0001
Word position	0.0227	0.0013	17.6020	<0.0001
Trial order	Removed			

**Random effects**	**Variance**	***SD***		

Word (Intercept)	0.0005	0.0224		
Participant (Intercept)	0.0022	0.0471		
Residual	0.0537	0.2317		

*L2RC = L2 reading proficiency; L2RSE = L2 reading self-efficacy; WF = word frequency, WL = word length, PWF = preceding word frequency, PWL = preceding word length, removed = removed during model selection.*

**FIGURE 1:**
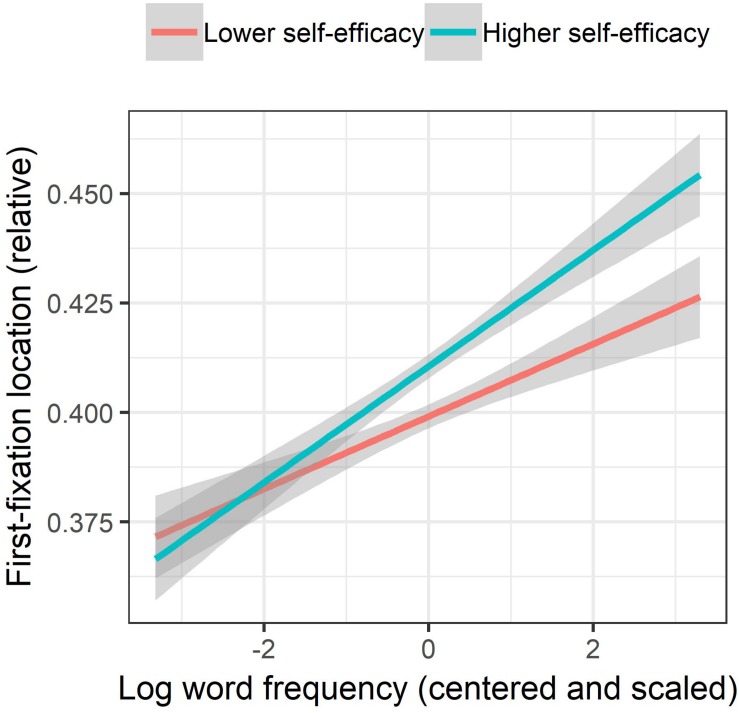
Interaction between L2 reading self-efficacy and word frequency on first-fixation location. While L2 reading self-efficacy was treated as a continuous variable in the linear-mixed effects models, it is categorized and displayed in two quantiles (Lower and Higher). Partial effects were obtained with the remef (Hohenstein and Kliegl, 2015) and ggplot2 packages (Wickham, 2009). Error bands indicate 95% confidence intervals.

**FIGURE 2:**
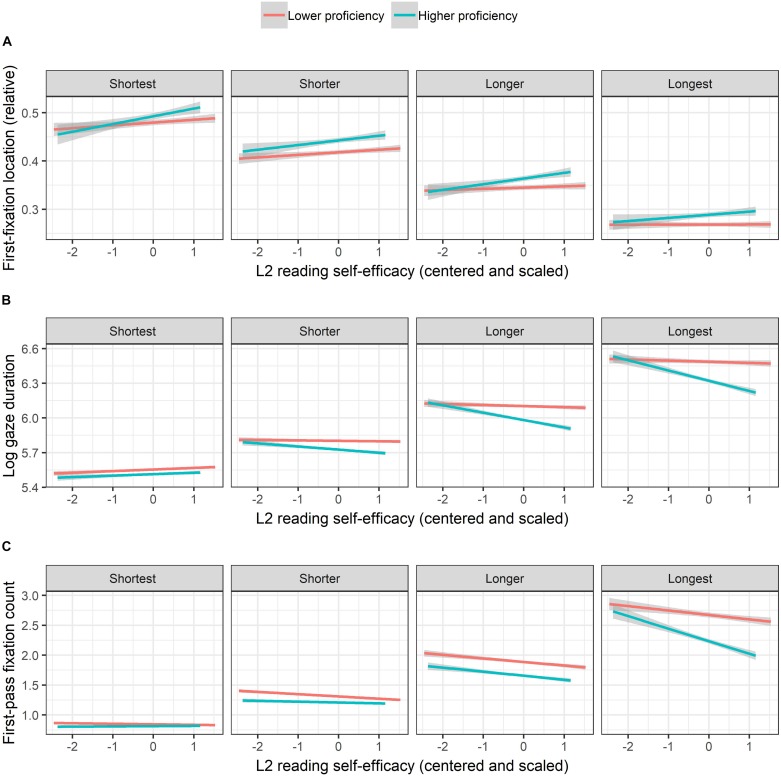
Interactions among L2 reading proficiency, L2 reading self-efficacy, and word length on first-fixation location **(A)**, gaze duration **(B)**, and first-pass fixation count **(C)**. While L2 reading proficiency and word length were treated as continuous variables in the linear-mixed effects models, they are categorized and displayed in two (Lower and Higher) and four (Shortest, Shorter, Longer, and Longest) quantiles, respectively. Partial effects were obtained with the remef (Hohenstein and Kliegl, 2015) and ggplot2 packages (Wickham, 2009). Error bands indicate 95% confidence intervals.

First, a significant two-way interaction between L2RSE and WF showed that the extent of the rightward shift of first-fixation location due to increase in WF was larger among more self-efficacious than less self-efficacious participants (Figure 1), indicating larger frequency effects with increasing L2RSE. Moreover, a significant three-way interaction among L2RC, L2RSE, and WL and an embedded two-way interaction between L2RSE and WL were observed. As illustrated in Figure 2A, while on average first-fixation locations shifted leftward with increasing WL, the extent of the change in first-fixation locations among more proficient participants was smaller than that among less proficient ones, when L2RSE was higher. In other words, for longer words, the effect of L2RSE on first-fixation location, indicating a positive relationship between the two variables (i.e., first-fixation location shifting rightward with an increase in L2RSE) was stronger among more proficient (i.e., fitted-lines showing a steeper slope) than among less proficient participants (i.e., fitted-lines showing a flatter slope).

### Gaze Duration

The results for the final model fitting gaze duration are displayed in Table 3. Similar to the results for first-fixation location, WF and WL significantly influenced gaze duration, indicating that on average, an increase in WF or a decrease in WL resulted in shorter gaze duration. A negative slope for L2RC was significant, indicating shorter gaze durations with increasing L2RC, while the main effect of L2RSE was not significant.

**TABLE 3:** Linear mixed-effects model fitting gaze duration.

	***b***	***SE***	***t***	***p***
**Fixed effects**				
Intercept	5.8660	0.0171	342.8640	<0.0001
L2RC	–0.0558	0.0152	–3.6750	0.0005
L2RSE	–0.0126	0.0154	–0.8190	0.4166
WF	–0.1143	0.0078	–14.6240	<0.0001
WL	0.3184	0.0076	42.0710	<0.0001
L2RSE × WF	Removed			
L2RSE × WL	–0.0164	0.0020	–8.3840	<0.0001
L2RC × WF	0.0065	0.0019	3.4070	0.0007
L2RC × WL	–0.0239	0.0019	–12.3930	<0.0001
L2RC × L2RSE	–0.0263	0.0162	–1.6220	0.1105
L2RC × L2RSE × WF	Removed			
L2RC × L2RSE × WL	–0.0130	0.0021	–6.3090	<0.0001
PWF	–0.0137	0.0029	–4.6640	<0.0001
PWL	Removed			
Word position	0.0122	0.0033	3.6580	0.0003
Trial order	0.0099	0.0019	5.1550	<0.0001

**Random effects**	**Variance**	***SD***		

Word (Intercept)	0.0221	0.1486		
Participant (Intercept)	0.0132	0.1150		
Residual	0.2023	0.4497		

*L2RC = L2 reading proficiency; L2RSE = L2 reading self-efficacy; WF = word frequency, WL = word length, PWF = preceding word frequency, PWL = preceding word length, removed = removed during model selection*

**FIGURE 3:**
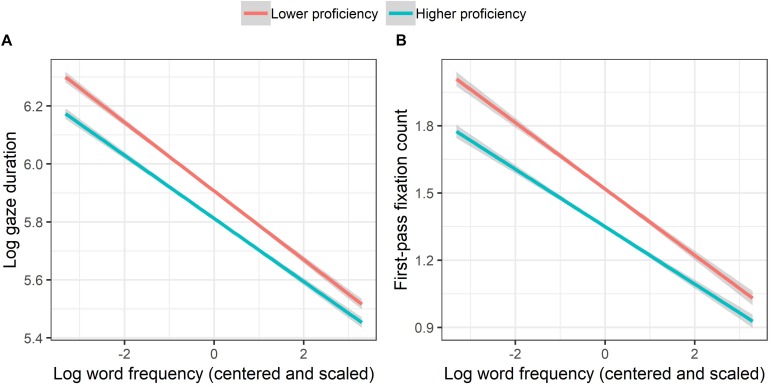
Interactions between L2 reading proficiency and word frequency on gaze duration **(A)** and first-pass fixation count **(B)**. While L2 reading proficiency was treated as a continuous variable in the linear-mixed effects models, it is categorized and displayed in two quantiles (Lower and Higher). Partial effects were obtained with the remef (Hohenstein and Kliegl, 2015) and ggplot2 packages (Wickham, 2009). Error bands indicate 95% confidence intervals.

L2RC interacted significantly with WF, showing larger WF effects among less proficient than among more proficient participants (Figure 3A). That is, while decrease in WF prolonged gaze duration, the extent of the prolongation was smaller with increasing L2RC. Importantly, a significant three-way interaction among L2RC, L2RSE, and WL, which embedded two significant two-way interactions, between L2RSE and WL and between L2RC and WL, was observed. As displayed in Figure 2B, while the increase in WL resulted in longer gaze duration on average, with increasing WL, gaze duration drop with increasing L2RSE was larger among more proficient than among less proficient participants. In other words, larger effects of L2RSE were observed with increasing L2RC for longer words.

### First-Pass Fixation Count

As shown in Table 4, the results for first-fixation count were similar to those for gaze duration. Significant main effects of WF, WL, and L2RC, were observed, indicating that increasing fixation count was associated with decreasing WF or L2RC, or with increasing WL. The main effect of L2RSE was not significant.

**TABLE 4:** Linear mixed-effects model fitting first-pass fixation count.

	***b***	***SE***	***t***	***p***
**Fixed effects**				
Intercept	1.4450	0.0285	50.6730	<0.0001
L2RC	–0.0994	0.0221	–4.5020	<0.0001
L2RSE	–0.0429	0.0224	–1.9190	0.0602
WF	–0.1393	0.0174	–8.0030	<0.0001
WL	0.5973	0.0168	35.4660	<0.0001
L2RSE × WF	Removed			
L2RSE × WL	–0.0385	0.0033	–11.4990	<0.0001
L2RC × WF	0.0117	0.0033	3.5880	0.0003
L2RC × WL	–0.0820	0.0033	–24.9050	<0.0001
L2RC × L2RSE	–0.0190	0.0236	–0.8040	0.4248
L2RC × L2RSE × WF	Removed			
L2RC × L2RSE × WL	–0.0217	0.0035	–6.1780	<0.0001
PWF	Removed			
PWL	–0.0197	0.0051	–3.8670	0.0001
Word position	–0.0284	0.0060	–4.7060	<0.0001
Trial order	Removed			

**Random effects**	**Variance**	***SD***		

Word (Intercept)	0.1154	0.3397		
Participant (Intercept)	0.0278	0.1667		
Residual	0.5925	0.7698		

*L2RC = L2 reading proficiency; L2RSE = L2 reading self-efficacy; WF = word frequency, WL = word length, PWF = preceding word frequency, PWL = preceding word length, removed = removed during model selection.*

L2RC interacted significantly with WF, showing larger WF effects among less proficient than among more proficient participants (Figure 3B). The difference in fixation counts between more proficient and less proficient participants grew with decreasing WF. Again, a significant three-way interaction among L2RC, L2RSE, and WL, which embedded two significant two-way interactions, between L2RSE and WL and between L2RC and WL, was observed (Figure 2C). While an increase in WL resulted in a higher fixation count on average, for longer words, fixation counts dropped more with increasing L2RSE among more proficient than among less proficient participants.

## Discussion

The present study aimed to investigate whether and how positive psychology is associated with L2 mental process during L2 reading behavior. We connected Fredrickson’s (2001) broaden-and-build theory with the dichotomous conceptualization of eye-movement patterns based on the dual-route word recognition model (Coltheart et al., 2001), with a view to expounding the mechanism underlying how positive belief toward L2 reading, indexed by L2 reading self-efficacy, might influence word reading strategies. We monitored the eye movements of a group of Japanese learners of English in a sentence reading task. Eye-movement patterns showing first-fixation locations closer to the beginning of a word were considered to show a preference for a local strategy attempting to process a word via the sublexical route, whereas first fixations located closer to the center of a word were considered to favor a global strategy for processing a word via the lexical route. Based on the broaden-and-build theory (Fredrickson, 2001), we anticipated that more self-efficacious L2 readers would prefer a global strategy during L2 reading.

The research questions were:

(1) Does L2 reading self-efficacy influence L2 reading strategies?
(2) How is L2 reading self-efficacy associated with L2 proficiency and word properties with respect to effects on reading strategies?

The present results showed that L2 reading self-efficacy modulated the effects of L2 reading proficiency and word properties on first-fixation location. Effects of L2 reading self-efficacy grew among readers with higher L2 reading proficiency for longer words, as well as for higher frequency words. Overall, the direction of the effect of L2 reading self-efficacy was that more self-efficacious L2 readers tended to have more first fixations positioned farther away from the beginning of a word. Hence, the present data provide a positive answer to research question 1. Previous findings supporting the broaden-and-build theory (Fredrickson, 2001) demonstrate that positive emotions broaden attentional scope during visual tasks (Fredrickson and Branigan, 2005; Wadlinger and Isaacowitz, 2006; Rowe et al., 2007); the present findings not only concur with these findings but also suggest that such an effect from positive psychology can also be observed in the mental processes during L2 behavior. Specifically, readers with more positive belief toward their L2 reading skills attend more to the global but not the local features of a word – as illustrated in the present study by participants with higher L2 reading self-efficacy being attracted to whole-word information over sublexical information such as letters and graphemes, whereas L2 readers with lower L2 reading self-efficacy attempted to glance the letters before reading the word, gathering fragmentary pieces of sublexical information to assemble the whole picture.

In the field of L2 studies, while positive psychology has attracted increasing interest among researchers, most of the studies have focused on how positive psychology is associated with L2 proficiency/L2 test performance (e.g., Lake, 2013; Dewaele and Alfawzan, 2018; Saito et al., 2018), as well as with L2 classroom behavior (Dewaele and Dewaele, 2018; Khajavy et al., 2018); little research has been done with L2 processing in mind. The present study provides fresh evidence that being positive toward one’s own L2 abilities affects moment-to-moment decision making in mind; that is, eye movements are modulated as a result of a tendency to broaden one’s attentional scope and target a fixation position closer to the center of a word, which brings more letters of a word into a visual area of highest acuity (i.e., the fovea).

Consistent with previous findings for first-language readers (Hawelka et al., 2010; Kuperman and Van Dyke, 2011), effects of both word length and word frequency on first-fixation locations were found significant, suggesting an increasing likelihood of preferring a global strategy to a local strategy for shorter or higher frequency words among our L2 participants. While the effect of language proficiency, which has been reported as a factor that influences strategic preferences in L1 readers (Kuperman and Van Dyke, 2011) and L2 readers (de León Rodríguez et al., 2016), was only marginally significant, the direction of the effect is similar to those reported in previous studies (Kuperman and Van Dyke, 2011; de León Rodríguez et al., 2016), with more proficient L2 readers favoring a global strategy. Importantly, word properties and L2 proficiency interacted with L2 reading self-efficacy, answering research question 2.

As shown by the significant interaction between word frequency and L2 reading self-efficacy, the present data indicate that the word frequency effect on reading strategy increases with higher L2 reading self-efficacy. Since the word frequency effect has been mostly interpreted as a learning effect (Brysbaert et al., 2018), this interaction effect between L2 reading self-efficacy and word frequency suggests both that L2 readers tend to employ a global strategy for words that have been repeatedly encountered and well learned before and that L2 reading self-efficacy increases the likelihood of such a strategic preference.

Regarding the three-way interaction involving word length, L2 proficiency, and L2 reading self-efficacy, while the present data did not identify a significant main effect of L2 reading proficiency, the three-way interaction reported indicates that such a proficiency effect not only depends on the length of an upcoming word but also on the extent of one’s positive beliefs toward one’s reading abilities. As shown in Figure 1A, the difference in first-fixation locations between less and more proficient L2 readers was larger when L2 reading self-efficacy was higher. This finding extends the literature by showing that the effect of L2 proficiency on preference of reading strategies, reported in de León Rodríguez et al. (2016), may not alone be enough to trigger a change in reading strategies. Viewing the interaction from another angle, the effects of L2 reading self-efficacy dropped when L2 reading proficiency was lower; this supports the notion that the effect of one’s self-efficacy depends on one’s skills and knowledge (Schunk and Pajares, 2002).

Nevertheless, the reason why the effects of L2 reading self-efficacy dropped with decreasing L2 reading proficiency is unclear. One plausible explanation is that less proficient participants in the present study lacked the requisite decoding skills, partly due to a lack of exposure to English (their L2), leading to a less detailed mental lexicon, which in turn tended to defer lexical route processing (Share, 1999). Another, related account involves allocation of visual attention during reading. The premise of the idea of moderating effects of word properties on first-fixation location for the upcoming word is that readers can preprocess part of the information from upcoming words in the parafovea (where visual acuity is lower than in the fovea; for review, see Rayner, 1998, 2009; Schotter et al., 2012) and that the extent of attention directed to upcoming words in the parafovea depends on reading skills (Rayner, 1986; Häikiö et al., 2009; Veldre and Andrews, 2014), reading speed (Rayner et al., 2010; Ashby et al., 2012; for L2 readers, see Leung et al., 2014), and exposure to target language (for bilingual and L2 readers, see Whitford and Titone, 2015). Based on this account, less skilled participants, who read more slowly (i.e., longer gaze duration) than more skilled ones, might have experienced difficulty utilizing information from longer words gathered in the parafovea in the present study. Future studies should further examine what kinds of L2 reading skills are associated with the effect of L2 reading self-efficacy on L2 reading processes, as well as how parafoveal processing is involved.

Regarding the results for gaze duration and first-pass fixation count, first, expected effects of word frequency, word length, and proficiency, as well as their interactions, are in line with previous findings on eye movements during reading (e.g., Joseph et al., 2009; Hawelka et al., 2010; Kuperman and Van Dyke, 2011; for L2 and bilingual readers, see Whitford and Titone, 2012). Importantly, similar to the results for first-fixation location, three-way interactions involving L2 reading proficiency, L2 reading self-efficacy, and word length were observed. All together, the present data on the three eye-tracking measures (see Figures 2A–C) suggest that self-efficacious L2 readers tend to optimize their reading strategy so as to enhance processing efficiency, preferring a more efficient global strategy more than readers of lower self-efficacy or lower reading proficiency do. This “efficiency” account of the effects of self-efficacy is in line with the notion of Maddux (2009, p. 339; see also Bandura, 1997) that “self-efficacy beliefs influence the efficiency and effectiveness of problem solving and decision-making.”

Pedagogically, the present findings provide a potential explanation of how pedagogical measures such as extensive reading improve L2 reading performance. Extensive reading is one of the most researched pedagogical measures for reading, and the bulk of empirical findings show that it has a positive influence on reading (see Nakanishi, 2015; Jeon and Day, 2016). Such a positive influence includes increasing reading rate (e.g., McLean and Rouault, 2017) and self-efficacy (Lake, 2014). In this regard, the present data may help explain how reading rate is enhanced via extensive reading by elucidating the relationship between word processing efficiency and self-efficiency, in light of reading strategy.

To conclude, the present study has shed fresh light on the effects of L2 reading self-efficiency on L2 reading strategy during sentence reading. The present data suggest that L2 reading self-efficacy interacts with other factors such as L2 reading proficiency and word properties to modulate reading strategies: as reflected by eye-movement measures, self-efficacious L2 readers tend to prefer a more global, more efficient reading strategy than those of lower self-efficacy, lending support to Fredrickson’s (2001) broaden-and-build theory in the context of L2 processing. The current findings are important. While positive psychology has drawn increasing interest among L2 researchers, research has been limited to classroom-based tasks and investigation (e.g., Dewaele and Dewaele, 2018; Khajavy et al., 2018), and studies employing laboratory-based tasks examining the relation between positive psychology and mental processes during real-time L2 behavior have been very few. The present study hence presents novel empirical evidence that positive psychology is associated with moment-to-moment decision making in L2 learners’ mind during L2 reading.

Several valuable implications of the present study can be identified. First, the study highlights the importance of the relation between positive psychology and L2 proficiency. While a more efficient global strategy is preferred with increasing L2 reading proficiency (de León Rodríguez et al., 2016), the present findings suggest that such a strategic change due to development of L2 proficiency may be hindered if emotional factors are not taken into consideration. An L2 reader may still prefer a less efficient local strategy to read words if the reader does not develop a positive belief toward his/her own reading abilities. In this regard, for education practitioners, teaching materials or classroom activities which might be too difficult for the students should be avoided, as enhanced self-efficacy is built from successful experiences (Bandura, 1997; Maddux, 2009). Thus, the aforementioned pedagogical measure, extensive reading, which stresses the important of pleasure reading, should be effective and feasible (Lake, 2014).

A methodological limitation of the present study is the correlational approach. While the current method also has its advantages, for instance regarding the size of the data set (the reading corpus) and the number of data observations (Angele et al., 2015), the effects of L2 reading self-efficacy arose mainly in the interaction effects. More studies, utilizing an experimental approach with stricter control of stimuli, are needed to refine the present findings in future.

## Data Availability Statement

The datasets generated for this study will be made available by the corresponding author upon reasonable request.

## Ethics Statement

Ethical review and approval was not required for the study on human participants in accordance with the local legislation and institutional requirements. All participants provided their written informed consent to participate in this study.

## Author Contributions

CL and LY collected the data. CL wrote the first draft. All authors contributed to the design and analysis of the data, and revised the drafts.

## Conflict of Interest

The authors declare that the research was conducted in the absence of any commercial or financial relationships that could be construed as a potential conflict of interest.

## Footnotes

1 First-fixation location is also termed “initial landing position.”

2 This example is excerpted from Coltheart (2005, p. 9).

3 Gaze duration includes words that received both single and multiple fixations.
